# Coping with Stress in Complicated Pregnancy and Gestational Weight Gain

**DOI:** 10.3390/ijerph181910493

**Published:** 2021-10-06

**Authors:** Agnieszka Rolińska, Anna Aftyka, Marzena Samardakiewicz

**Affiliations:** 1 Chair and Department of Psychology, Faculty of Medicine, Medical University of Lublin, 20-059 Lublin, Poland; marzena.samardakiewicz@umlub.pl; 2 Department of Anesthesiological and Intensive Care Nursing, Faculty of Health Sciences, Medical University of Lublin, 20-059 Lublin, Poland; anna.aftyka@umlub.pl

**Keywords:** coping with stress, gestational diabetes, pregnancy, excessive weight gain

## Abstract

Maternal obesity is one of the leading health problems in the world. Excessive gestational weight gain (GWG) can lead to many complications during pregnancy, especially when it is accompanied by diabetes. Moreover, the risk of excessive GWG in pregnant women is significant, irrespective of prenatal counseling. Studies on this subject concerning coping with stress are lacking in the literature. The present work is aimed at evaluating the styles of coping with stress and their relation to GWG in pregnant women with gestational diabetes (GDM) for whom sudden adaptation to dietary management during this period can be challenging. It was indicated that women with GDM reported high stress related to potential maternal-fetal complications and worries about compliance with dietary management. The overall weight gain of participants in pregnancy was determined in connection to their prepregnancy body mass index (BMI) and classified based on the Institute of Medicine guidelines. A standardized psychological scale was used to assess coping styles. The results showed that almost half of the participants did not meet the Institute of Medicine recommendations for weight gain during pregnancy. There were significant correlations between the styles of coping with stress and the GWG. Additionally, low correlations were indicated between emotional, avoidant, task-oriented coping styles and the age of pregnant women with GDM. Regression analysis showed that the stress-coping style that focused on emotions was the most predictive of overall weight gain. There is a need for a better understanding of psychological barriers in achieving the recommended GWG and potential limitations in providers’ interventions, particularly for GDM.

## 1. Introduction

Maternal obesity is a growing public health concern in the world, linking to gynecological and obstetrical complications during pregnancy [1,2]. Excessive gestational weight gain (GWG) is a significant contributor to the global obesity epidemic, associated with increased risks of maternal, fetal, and childhood negative health outcomes [3,4]. Gestational weight gain (GWG) mainly reflects the maternal nutritional status as well as tissue expansion during pregnancy because of fat storage, and the presence of amniotic and extracellular fluids [5]. According to the literature, up to 70% of women gain a higher weight than that recommended by the Institute of Medicine [6,7,8,9]. One of the very common social standards during this period can be summarized by the frequently used phrase “eating for two”. Lots of women describe pregnancy as a time to gain weight freely without criticism [10]. Moreover, pregnancy is characterized by insulin resistance and hyperinsulinemia, which increases the risk of the development of diabetes in some women. During pregnancy, placental secretion of diabetogenic hormones such as growth hormone, glucagon, cortisol, human placental lactogen, and progesterone leads to insulin resistance [11]. Diabetes is the most common metabolic disorder of pregnancy. The International Diabetes Federation suggests that 16.8% of pregnancies are affected by diabetes and the majority (86.4%) are affected by gestational diabetes mellitus (GDM) [12]. In diabetics, carbohydrate metabolism is controlled by implementing a suitable diet, insulin therapy, and/or oral medications. Diet is the basis of interventions in every type of diabetes. Stress and anxiety were reported when women with gestational diabetes talked about following dietary advice [13]. Compliance with recommended weight gain during pregnancy has a significant impact on the fetal outcome in pregnant women with gestational diabetes [14]. In women with GDM, excessive GWG has been shown to increase the incidence of cesarean section and infant macrosomia compared with women whose GWG is within the Institute of Medicine recommendations [15].

A qualitative study that analyzed the attitudes of physicians and nurse midwives toward weight gain in pregnancy showed that providers believed that counseling had only a low impact on pregnant women and that patients were more influenced by other factors, such as family, habits, and culture, and therefore, they avoided counseling due to the sensitivity of the topic [16]. During pregnancy, focusing on weight management can be beneficial for both the mother and her infant; however, promotion and education are very challenging. Psychological context should be acknowledged to achieve better care for the future. From the perspective of programming of obesity and metabolic function, individuals exposed to prenatal stress during pregnancy have higher BMI and primary insulin resistance later in their life [17]. During pregnancy, stress can be caused by dietary patterns and physical activity. According to Lobel et al. (2008), pregnancy-specific stress is directly associated with unhealthy eating and inversely associated with healthy eating and exercise if controlled for obstetric risk [18]. This association is not just limited to a healthy pregnancy. Hui et al. (2014) reported that in GDM, unhealthy diet-coping strategies were linked with anxiety and stress [19]. Another study indicated that in complicated pregnancy, perceived stress was significantly related to physical activity [20]. Some results showed that women with lower stress levels frequently achieved the recommended GWG [21], however, other data did not confirm this association [22]. The study, by Denisoff and Endler (2000) focused on the relationships among stress, styles of coping with stress, and weight preoccupation in women [23]. The authors showed that emotion-oriented coping predicted weight preoccupation regardless of stress in a group of 206 females (aged 19–55 years). Variability in coping behaviors or abilities can be related to differences in the physiological influence of stressors on pregnant women [24]. Emotion-focused coping was negatively correlated in pregnancy with physical functioning sub-dimensions of the 36-Item Short-Form Health Survey (SF-36) [25]. The results of path analysis by Dolatian et al. (2020) indicated that among intermediate determinants of health, symptoms of stress, anxiety, depression, pregnancy-specific stress, and violence had an indirect effect on weight gain in pregnancy [26].

A review of the literature has been conducted to identify articles that reported studies on the relationship between coping with stress and weight gain in pregnancy, using the databases: PubMed/Medline, Academic Search Complete (EBSCOhost), APA PsycArticles (EBSCOhost), and Studies on Women & Gender Abstracts. The inclusion criteria were published: in English, in a peer-reviewed journal, and/or academic journal. Each database was initially searched using the following terms: weight gain OR gestational weight gain OR pregnancy weight gain OR gwg AND coping OR cope OR stress coping OR coping with stress OR coping strategies OR coping skills OR coping styles AND gestational diabetes OR GDM OR gestational diabetes mellitus OR diabetes in pregnancy. In the PubMed and EBSCO databases, the medical subject headings (MeSH terms) were used: “Stress”, “Stress, Psychological”, “Gestational Weight Gain”, “Weight Gain”, “Pregnancy”, “Diabetes Mellitus”. Additionally in EBSCO subject terms were also checked: weight gain, pregnancy outcomes, pregnancy complications, diabetes, gestational diabetes, psychological stress. Three studies have been found. Only one of the studies considered this relationship in pregnancy complicated by diabetes [27]. The relationship between GWG and stress coping in pregnancy has been underestimated, especially in pregnant women with diabetes mellitus. The present study has been aimed at analyzing the stress coping styles as the potential psychosocial risk factor for excessive GWG in women with GDM. 

## 2. Materials and Methods

### 2.1. Participants and Procedure

The research project of our study was approved by the Bioethical Commission at the university where the study was conducted. The data were obtained from outpatient hospital gynecological-obstetrical clinics and the Department of Obstetrics and Pathology of Pregnancy-Public University Hospital No. 1 in Lublin, and the gynecological-obstetrical outpatient clinic in one of the medical centers in Rzeszów, between June 2014 and December 2016. Participation in the study was voluntary. After examining the objective and procedures of the research project, the patients declared their willingness to participate. Written informed consent was obtained from all participants. The inclusion criteria for participation in the study were as follows: single pregnancy, primogeniture, and no serious underlying conditions (except for GDM, and possible pregnancy-induced hypertension, which is often comorbid with diabetes). Of all the women invited to participate in the study, who were screened for GDM after diagnosis of pregnancy or between 24–28 weeks of pregnancy, 200 agreed to take part, fulfilled a standardized psychological scale, and reported their anthropometric data (weight, height, prepregnancy body mass index (BMI)). During the first stage of the study, sociodemographic data of the respondents (age, education, place of residence, socioeconomic status, and family history of diabetes) were also collected. In the second stage of the study, participants’ GWG and a gestational week at delivery were checked. Women had underweight, twin pregnancy, miscarriages, preterm birth (before 37 completed weeks of gestation), changed hospital or clinic, were on insulin therapy (not only carbohydrate-controlled diet), gave insufficient information on their: pre-pregnancy BMI and/or prepregnancy weight, and height or other missing data were excluded. The final sample for analysis included 102 women with GDM. 

### 2.2. Measures

Gestational diabetes was diagnosed in the study group by performing a fasting 75-g oral glucose tolerance test. The results were interpreted based on the levels of venous plasma glucose before and 2 hours after a 75 grams oral glucose load.

Body mass index or BMI is a statistical index using a person’s weight and height to provide an estimate of body fat in males and females of any age. It was calculated by taking a person’s weight, in kilograms, divided by their height, in meters squared (BMI = weight (in kg)/height (in m)^2^ [28]. GWG data were obtained by comparing self-reported prepregnancy BMI or BMI during 1–3 weeks of pregnancy with the weight measured before delivery. The data received were evaluated concerning the Institute of Medicine (IOM) recommendations for prepregnancy BMI categories. The recommended amount of GWG is 11.5–16 kg, 7–11.5 kg, and 5–9 kg for women entering pregnancy with normal BMI (18.5–24.9 kg/m^2^); overweight (25–29.9 kg/m^2^) and obese (≥30 kg/m^2^), respectively [29]. The participants were divided into two groups based on their total GWG: appropriate GWG (within the IOM recommendations), inappropriate GWG (beyond the IOM recommendations). 

The Coping Inventory for Stressful Situations (CISS) was used to collect psychological styles of coping. CISS Inventory, developed by Endler and Parker [30,31], was derived from both theoretical and empirical bases and has frequently been used in a variety of research focusing on styles of coping with stress. Coping styles refers to more consistent tendencies to cope in particular ways. The Inventory consists of 48 items and allows identifying three styles of stress coping: task-oriented style, emotion-oriented style, and avoidance-oriented style. Items are scored on a 5-point Likert scale (from 1 not at all to 5 very much). Scores for all items per scale are summed to form scale scores. The minimum score in each of the scales is 16, the maximum—80 points. Higher scores indicate greater use of that particular coping strategy. The emotion-oriented subscale describes emotional reactions that are self-oriented (e.g., get angry, blaming oneself, become tense, or upset). Task-oriented coping emphasizes attempts to solve the problem. The avoidance-oriented coping strategy may be employed by making use of one’s social networks or by distracting oneself through engaging in self-rewarding activities like shopping and eating. This diagnostic tool has already been used in the population of pregnant women [32,33]. A Polish version of the questionnaire is showed a satisfactory internal consistency (coefficients between 0.74 and 0.88) and stability for the main three scales (correlations with a retest done 2–3 weeks later—coefficients between 0.75 and 0.80), as well as a confirmed factor, and theoretical and criterion validity [34].

The authors’ questionnaire was used to collect sociodemographic data of the respondents (age, education, place of residence, socioeconomic status, and family history of diabetes) and self-reported anthropometric data. 

Data were analyzed using the SPSS software (v. 13.0, SPSS Inc., Chicago, IN, USA). Quantitative variables were characterized by descriptive statistics such as maximum and minimum, mean, median, and standard deviation. The normality of the distribution of quantitative variables was assessed using the Shapiro–Wilk test. The relationship between the studied variables was evaluated using correlation coefficients. Regression analysis was performed by the stepwise regression method with a backward-elimination approach. This modeling technique aims to maximize the prediction power with a minimum number of predictor variables.

## 3. Results

The mean gestational week of the participants at inclusion was 25.8 ± 1.28 and at delivery was 38.7 ± 1.27. Their mean age was 28.92 ± 4.45 years. The sociodemographic and clinical characteristics of the participants are presented in Table 1.

The descriptive statistics for weight gain and stress coping styles—emotion-, avoidance-, and task-oriented—analyzed for individual groups were presented in Table 2.

Of the analyzed women, 102 pregnant women participated in the study. Among them, 43 (42.16%) gained higher weight, above the IOM recommendations, and 49 (58.04%) gained the recommended weight. GWG of the study group (*n* = 102) ranged from 5.8 kg to 25 kg. The mean weight gain of the whole group from conception until delivery was 13.02 ± 4.07 kg. Significant differences in weight gain were found (S = 32.86, *p* < 0.001)—it was the highest in the group of women with normal body weight (14.79 ± 3.44 kg), slightly lower among overweight women (12.16 ± 3.88 kg), and the lowest—in women with obesity in the period before pregnancy (8.03 ± 2.10 kg)—Table 2. In the study group, prepregnancy BMI values were found to be in the normal range for 49 women, in the overweight range for 43 women, and in the obese range for 10 women (type I obesity). There was found a significant moderate negative correlation between pre-BMI and GWG (rho = −0.48, *p* < 0.00) There wasn′t any significant association between maternal age and GWG.

In the whole sample, the highest mean was indicated for task-oriented coping (52.39 ± 16.40 kg), emotional coping (46.25 ± 16.97), avoidant coping (44.73 ± 15.53), respectively. Low correlations were indicated between emotional, avoidant coping and age (rho = −0.20, rho = −0.28; *p* < 0.00), and between task-oriented coping and age (rho = 0.23, *p* < 0.00). There was not reported any significant association between styles of coping and pre-BMI. A very strong correlation was reported between emotional and avoidant coping styles (rho = 0.92, *p* < 0.01). The weight gain in kilograms of the respondents was on average positively correlated with the emotional (rho = 0.47, *p* < 0.001) and avoidant (rho = 0.46, *p* < 0.001) styles of coping with stress. This means that greater body weight was observed in women who extensively used these stress coping styles. Moderate negative correlations were found between the weight gain of the participants and the use of task-oriented coping style (rho = −0.40, *p* < 0.01)—Table 3.

The next step in the research was regression analysis using backward stepwise regression, which involves starting with all candidate variables, testing the deletion of each variable using a chosen model fit criterion, deleting the variable (if any) whose loss gives the most statistically insignificant deterioration of the model fit, and repeating this process until no further variables can be deleted without a statistically insignificant loss of fit. All statistically significantly numerical variables correlated with GWG (task-oriented coping, emotional coping, avoidant coping) were included in the analysis. Regression analysis performed in the study group was shown that the strongest predictor of weight gain in kilograms was emotional coping. This variable is explained as 22.5% of the variance in the above-discussed variable (R^2^ = 23.3%, R^2^ adjusted = 22.5%, *p* < 0.001)—Table 4.

## 4. Discussion

In the available literature, some studies have focused on the genetic determinants of weight gain in pregnancy [35]. However, unlike the biological or demographical factors, psychological determinants seemed to be potentially modifiable in the context of weight gains [36]. Considering the important role of education, some data suggest that women who had adequate or inappropriate GWG received comparable prenatal counseling on lifestyle modifications, weight gain, and nutrition [37]. Moreover, pregnant women who believed that external factors mainly determine fetal health appeared to be more nonadherent to clinical GWG guidelines [38]. Hill et al. (2016) noticed that a better understanding of the psychological aspects of weight gain allowed for more effective planning of intervention strategies in the case of pregnancy [39]. It is worth mentioning that planning appropriate psychological and behavioral interventions to maintain appropriate GWG is a challenge [40,41,42,43], particularly for women coping with GDM who manifested stress, anxiety, and worries to potential complications, as well as frustration and anger when advised to change long-standing eating behaviors and habits [44]. Additionally, ineffective coping styles with stress are at higher levels in pregnant women with GDM compared to women in non-high-risk pregnancies [45]. 

In the present study, almost half of the participants (42%) have not been found to meet the IOM recommendations and gained excessive gestational weight. This finding is concerning and is also consistent with the results of other authors [6,7,8,15,46]. The mean overall weight gain (GWG) has been the highest in the women who had a normal preconception BMI, lower in overweight women, and the lowest in obese pregnant women. In the studied sample, a significant moderate, inversive correlation between pre-BMI and GWG was also indicated. In the study of other authors, it was reported that some physical factors, including prepregnancy BMI, might play a significant role in excess weight gain. Kheirouri S. et al. (2017) demonstrated a significant negative correlation between a mother’s prepregnancy BMI and maternal GWG [47]. Moreover, the analysis of the data of about 2.3 million singleton pregnancies also showed a negative correlation between BMI and maternal weight gain [48]. Pre-pregnancy overweight combined with EGWG leads to an increased risk of macrosomia, delivery complications, and even a higher long-term likelihood of remaining overweight or obese [49,50].

In the whole sample, the highest mean was indicated for task-oriented coping (52.39 ± 16.40 kg), emotional coping (46.25 ± 16.97), avoidant coping (44.73 ± 15.53), respectively. However, the differences between the mean weren’t high. In high-risk pregnancies, women probably are not only in strong stress and frustration against clinical restrictions related to the management of medical conditions but also concentrate on active coping due to the health of the fetus. Two correlations of a similar nature and strength were found, the weight gain in kilograms in the respondents was on average, positively correlated with the emotional and avoidance style of coping with stress. A moderate negative correlation was found between the weight gain of the respondents and the use of a task-oriented style. Additionally, a very strong correlation was indicated between emotional and avoidant styles of coping. Some authors consider avoidance coping as a form of emotion-focused coping [51]. Any relationships between styles of coping and prepregnancy BMI in women with gestational diabetes were not found. The strongest correlation was noticed between the emotional style of coping and GWG. In the subject literature higher use of emotion-oriented coping strategies was associated with a higher frequency of depressive moods during pregnancy [33]. Moreover, Varescon et al. (2013) reported that pregnant women who smoked during pregnancy use more emotion-focused coping than pregnant non-smokers [52]. 

The regression analysis indicated that the strongest predictor of gestational weight gain in kilograms in the presented sample was the use of an emotion-focused style. This variable explained 22.5% of the variance in gestational weight gain. The results of this study suggest that emotional coping can be potentially related to unhealthy eating behaviors. In a study conducted in a group of previously overweight women during the third trimester of pregnancy, the authors reported that difficulties in emotion regulation are associated with dysfunctional eating behaviors [53]. Furthermore, excessive GWG was significantly and positively correlated with emotional eating [54]. Emotional eating is defined as the consumption of food in response to emotional cues, even when not experiencing physical hunger [55]. This can be considered as a maladaptive coping strategy to relieve negative emotions. Raspopow et al. (2013) observed that emotional coping mediated the relationship between emotional eating and lack of support [56]. Moreover, some authors have reported that in females, emotion-focused coping partially mediated the relationship between stress and binge eating [57]. The pattern of weight gain in pregnancy complicated by binge eating has been shown to differ from that of normal pregnancy [58]. Additionally, McDonald S. D. et al. (2020) found that excess pregnancy weight gain was moderately predicted by nine psychological, physical, and social factors, including also having emotion control difficulties [59].

We reviewed the available literature using databases Medline, EBSCO, and Studies on Women & Gender Abstracts and found three studies corresponding with the theme of their research. One study based on correlations was conducted in a group of women with GDM [27]. The results indicated that the self-confident approach, as one of the effective coping styles, showed a significant negative linear relationship with weight gain during pregnancy (*r* = –0.342, *p* < 0.01) in 126 women with GDM. In the other two studies, the authors used regression analysis for assessing the relationship between GWG and stress coping styles; however, both were conducted on healthy pregnant women. The first study was conducted in a group of 225 women and focused on two forms of coping (planning–preparation and avoidance which were assessed by the Revised Prenatal Coping Inventory [60]. Using classification and regression tree analysis, the authors found that the above-mentioned factors were not significant predictors of excess GWG. The second study used a baseline and revised model (using logistic regressions and path analysis), in which pregnancy-specific and nonspecific coping (measured by the Revised Prenatal Coping Inventory and the COPE) were found to be potential predictors of excessive GWG, together with some other psychological, demographic, motivational, and behavioral variables [39]. However, the authors did not consider coping as an individual risk factor in this relationship, and their sample comprised mostly women of high socioeconomic status. It is also worth mentioning that some authors identified the styles of stress coping to be less effective in pregnancy complicated by GDM than in uncomplicated pregnancy [45].

The findings of the present study must be taken into account within the context of its limitations. Firstly, considering the methodological aspect of the study, it should be noted that information on the prepregnancy weight of participants was self-reported. According to some authors, self-reported weight tends to be underestimated which might lead to some potential misclassification [61]. However, a very high correlation has been found between the prepregnancy weight determined using questionnaires and the weight measured at patient intake (0.95, *p* < 0.001) based on the data obtained from them for research. In addition, according to other authors, self-reported prepregnancy weight generally correlated well with measured prepregnancy weight [62]. Although, most of the research conducted so far on the association between GWG and psychosocial factors has been based on self-reported pre-BMI. Secondly, we conducted only two stages of the study with one measurement of total GWG before delivery. We have not reported the weight gain in pregnant women in the first and the second trimester. Analyzing the effect of mental health and psychological functioning on pregnancy complications is very important however the relatively small sample size (102 participants) limited the statistical power of the conducted study. Furthermore, the study included only primiparous and pregnant women with GDM who were following a carbohydrate-controlled diet (without insulin therapy). These characteristics may limit the generalizability of the findings. Moreover, factors such as being multiparous or the type of diabetes treatment may be important in the relationship between weight gain and coping with stress in pregnancy. Some authors observed that multiparous women had a lower risk of excessive GWG, as compared to primiparous women [61,63]. Therefore, in further analysis, it is worthy to include participants treated by insulin therapy, with more balanced primiparous and multiparous proportions. 

## 5. Conclusions

Women in pregnancy complicated by diabetes can experience a range of physical and psychological challenges. In the presented study, a considerable number of women with GDM have not met the IOM standards for adequate gestational weight. Emotional style coping has been shown as the most predictive of gestational weight gain in kilograms in women with gestational diabetes. Additionally, low correlations were indicated between emotional, avoidant, task-oriented coping styles and the age of pregnant women with GDM. In the future, the relationship between longitudinal measurement of weight gain throughout pregnancy and coping with stress should be analyzed in normal and complicated diabetes pregnancy. Identifying women at high risk of excess pregnancy weight gain relatively early in pregnancy can allow more targeted interventions. Psychological screening may help in explaining the potential barriers to physicians’ and midwives’ interventions. 

## Figures and Tables

**Table 1:** Characteristics of the participants with gestational diabetes (*n* = 102).

		Total Group	Pre-BMI Normal (*n* = 49)	Pre-BMI Overweight (*n* = 43)	Pre-BMI Obesity (*n* = 10)	Test	*p*
Age (years); M (SD)		28.92 (4.45)	28.65 (4.91)	29.49 (3.91)	27.80 (4.34)	H = 1.799	0.407
Gestational week at inclusion; M (SD)		25.81 (1.28)	25.98 (1.32)	25.47 (1.14)	26.50 (1.35)	H = 6.152	0.046
Gestational week at delivery; M (SD)		38.70 (1.27)	38.63 (1.20)	38.91 (1.38)	38.70 (1.16)	H = 0.820	0.664
Education; *n* (%)	Vocational * Secondary or postsecondary ** Higher ***	19 (18.74) 45 (43.86) 38 (37.40)	12 (24.49) 17 (34.69) 20 (40.82)	4 (9.30) 21 (48.84) 18 (41.86)	3 (30) 7 (70) 0 (0)	Χ^2^ = 10.45	0.033
Place of residence *n* (%)	Rural Urban	40 (39.21) 62 (60.78)	18 (36.73) 31 (63.27)	17 (39.53) 26 (60.47)	5 (50.00) 5 (50.00)	Χ^2^ = 0.616	0.735
Subjective socioeconomic status; *n* (%)	Low Medium High	14 (13.74) 56 (54.86) 32 (31.40)	14 (13.74) 56 (54.86) 32 (31.40)	6 (12.24) 27 (55.10) 16 (32.65)	2 (20.00) 5 (50.00) 3 (30.00)	Χ^2^ = 0.467	0.976

M—mean, SD—standard deviation, H—Kruskal–Wallis H test, Χ^2^—chi-squared test, BMI—body mass index, Education: * Vocational (usually 13 schooling years), ** Secondary or postsecondary (usually 13–16 schooling years, often with A-level certificate), *** Higher (usually 16–18 schooling years, bachelor’s or master’s degree).

**Table 2:** Weight gain and styles of coping in the sample (*n* = 102).

		Min.	Max.	M	SD	Me
All samples (*n* = 102)	GWG (kg)	6	25	13.02	4.07	12.3
	Emotional coping (pt)	18	80	46.25	16.97	43.5
	Avoidant coping (pt)	17	80	44.73	15.53	42
	Task-oriented coping (pt)	20	80	52.39	16.40	54.5
Pre-BMI normal (*n* = 49)	GWG (kg)	9	25	14.79	3.44	13.2
	Emotional coping	18	80	48.14	15.57	48
	Avoidant coping (pt)	17	80	47.33	15.87	45
	Task-oriented coping (pt)	20	78	49.22	16.86	53
Pre-BMI overweight (*n* = 43)	GWG (kg)	7	23	12.16	3.88	11
	Emotional coping (pt)	18	80	43.30	17.21	42
	Avoidant coping (pt)	19	79	41.49	13.95	39
	Task-oriented coping (pt)	24	80	56.33	15.17	57
Pre-BMI obesity (*n* = 10)	GWG (kg)	6	11	8.03	2.10	7.25
	Emotional coping (pt)	26	79	49.70	21.97	40.5
	Avoidant coping (pt)	26	68	45.90	19.12	36
	Task-oriented coping (pt)	22	69	51.00	17.28	50.5

Min.—minimum, Max.—maximum, M—mean, SD—standard deviation, Me—median, BMI—body mass index, GWG—gestational weight gain, kg—kilogram, pt—point (CISS Inventory: min—16, max—80).

**Table 3:** Spearman’s rank correlation matrix for the whole group (*n* = 102).

	Age	Pre-BMI	GWG	Task-Oriented Coping	Emotional Coping
Age	x				
Pre-BMI	0.03	x			
GWG	−0.13	−0.48 *	x		
Task-oriented coping	0.23 *	0.11	−0.40 *	x	
Emotional coping	−0.20 *	−0.07	0.47 *	−0.77 *	x
Avoidant coping	−0.28 *	−0.11	0.46 *	−0.76 *	0.92 *

BMI—body mass index, GWG—gestational weight gain, * *p* < 0.05, statistically significant.

**Table 4:** Analysis of the backward stepwise regression in pregnant women with gestational diabetes (*n* = 102).

Model	Unstandardized Rates		Standardized Rates	t	*p*
	B	SE	β		
Constant	7.660	1.035		7.399	<0.0001
Emotional coping	0.116	0.021	0.482	5.508	<0.0001

SE—standard error, B—unstandardized regression coefficient, β—standardized regression coefficient, t—*t* test.

## Data Availability

The data presented in this study are available on request from the corresponding author. The data are not publicly available due to legal issues.
